# The Correlation Between the Immune and Epithelial-Mesenchymal Transition Signatures Suggests Potential Therapeutic Targets and Prognosis Prediction Approaches in Kidney Cancer

**DOI:** 10.1038/s41598-018-25002-w

**Published:** 2018-04-26

**Authors:** Jiayu Liang, Zhihong Liu, Zijun Zou, Yongquan Tang, Chuan Zhou, Jian Yang, Xin Wei, Yiping Lu

**Affiliations:** 1 Department of Urology, Institute of Urology, West China Hospital, Sichuan University, Chengdu, 610041 Sichuan China; 2 Center of Growth, Metabolism, and Aging, Key Laboratory of Bio-Resources and Eco-Environment, College of Life Sciences, Sichuan University, Chengdu, 610064 Sichuan China

## Abstract

Both epithelial-mesenchymal transition (EMT) and immune regulation are important biological process in malignant tumours. The current research aims to comprehensively explore the potential association between the epithelial-mesenchymal transition (EMT) signature and immune checkpoint signature and its role in predicting the prognosis of clear-cell renal cell carcinoma (ccRCC) patients. EMT-related genes were collected from an experiment-based study and then were investigated using data from the Cancer Genome Atlas. A total of 357 genes were included, and 23 of them that were upregulated and correlated with prognosis were analysed further as core EMT genes in ccRCC. Interestingly, the emerging immune checkpoints CD276, OX40 and TGFB1 were found to be significantly co-expressed with core EMT genes, and TGFB1, CXCR4, IL10, and IL6 were the most important molecules potentially interacting with EMT molecules in our model, as determined from mRNA co-expression and protein-protein interaction network analysis. Additionally, an integrated scoring model based on FOXM1, TIMP1 and IL6 was successfully established to distinguish ccRCC patients with different clinical risks. Our results identified core genes in the EMT-immunophenotyping correlation and evaluated their risk assessment capabilities, providing more potential therapeutic targets and prediction approaches regarding the translational research of treatment and prognosis in ccRCC.

## Introduction

A total of 14400 kidney cancer-related deaths were estimated to occur in the United States in 2017^1^. In China, the figure was estimated to be 23400 in 2015^2^. Clear-cell renal cell carcinoma (ccRCC) is the most common subtype of kidney cancer in adults, accounting for approximately 70% of all cases. Despite advances in diagnosis and treatments, approximately 20–30% of RCC is diagnosed in the metastasis stage, and another 20–30% of patient undergoing curative surgery for a primary tumour develop metastasis with poor prognosis during follow-up^3–5^. After the development of cytokine-based therapies and targeted therapies, the emerging immune checkpoint inhibitors (ICIs) are now becoming representative novel therapies for non-surgical treatment of RCC.

In the tumour microenvironment, immune checkpoint receptors negatively regulate the proliferation and activity of T cells and other immune cells and may induce immunosuppression and immunity escape. As reported by Liu XD *et al*., immune cell infiltration, especially increased T-lymphocyte infiltrates, is associated with poor survival of RCC patients^6^. However, although the advent of ICIs has revealed novel therapies for ccRCC, only a subset of individuals would benefit from the current PD-1, PD-L1, and CTLA-4 inhibitors due to no-response and resistance in RCC patients^7^.

Epithelial-mesenchymal transition (EMT) is a critical process in the progression, invasion and metastasis of a wide range of carcinomas. The expression of EMT-associated signatures is significantly correlated with metastasis and the prognosis of RCC patients^8,9^. In cancer cells, an abnormal EMT signature is linked not only to migration and invasion but also to various acquired capabilities, such as resistance to chemotherapy and immunotherapy alterations in DNA repair^10^. Recently, a new pan-cancer EMT signature was found to correlate with immune cell signalling according to Mak MP *et al*., offering new insights into the potential significance of the correlation between EMT and immune checkpoints^11^. Given that ccRCC is a predominantly mesenchymal tumour, the selection of its candidate EMT genes and the exploration of its correlation with the immune signature would be different from other tumour types. Thus, the present research aims to specifically explore the relationship between core molecules in EMT and immune targets in ccRCC, as well as the clinical significance of this correlation.

Here, we collected data from an experiment-based EMT gene set (dbEMT)^12^. A total of 23 core EMT-associated genes (CEGs) were found to be over-expressed and associated with prognosis in ccRCC using publicly available genomics resources. A subset of the signalling pathways involved in the EMT network were further identified to be linked to immune activities. Based on the potential EMT-immunophenotyping correlation mechanism, we comprehensively explored the association and interaction between CEG and selected targetable immune checkpoint molecules from different aspects, including the mRNA co-expression and protein-protein interaction (PPI) network. Subsequently, the two signatures were successfully integrated, and a clinical risk scoring system was established.

## Results

### Screening Core EMT-associated Genes (CEG) in ccRCC

A total of 357 EMT-associated genes were analysed (for further details of the ccRCC cohort, see Supplementary Table S1), and according to the criteria, 100 of the 357 (28.01%) genes were found to be differentially expressed. Among these, the expression of 46 (12.89%) genes were upregulated, and that of 54 (15.13%) was down-regulated (Supplementary Table S2).

We next investigated the correlation between the expression of the EMT signature and clinical outcomes, including the overall survival or disease-free survival status (The Cox proportional hazard ratio and 95% confidence interval were also included in the survival plot; see Supplementary Fig. S1). We observed that 126 (35.29%) EMT-associated genes were significantly associated with better patient outcomes. By contrast, the mRNA levels of 43 (12.04%) genes were negatively correlated with a favourable prognosis (Supplementary Fig. S2). The large number of genes influencing the prognosis indicated non-negligible functions of the EMT signature in ccRCC.

Considering that the biological influence of upregulated protein-coding genes was more directly observed, we merged the expression data and survival data in the following research, and 23 genes that were over-expressed and related to prognosis were defined as core EMT genes (CEGs) in our study (Supplementary Table S2). Among them, 14 genes were associated with the poor survival of ccRCC patients (Supplementary Fig. S1a), namely, TGFB1, LAMA5, PTHLH, FOXM1, TIMP1, CAV1, CDKN2A, ITGA5, CTSZ, LOX, PLAUR, MMP9, LOXL2 and FSCN1; the other 9 genes were related to a better prognosis (Supplementary Fig. S1c). In addition, the differentially expressed levels of CEGs were extracted and validated from GEO datasets (GSE53757), among which the remarkably higher expression of the entire 23 genes could be observed in tumour tissue (P < 0.05, Supplementary Table S3).

### Testing the clinical significance of CEG alteration

**Figure 1:**
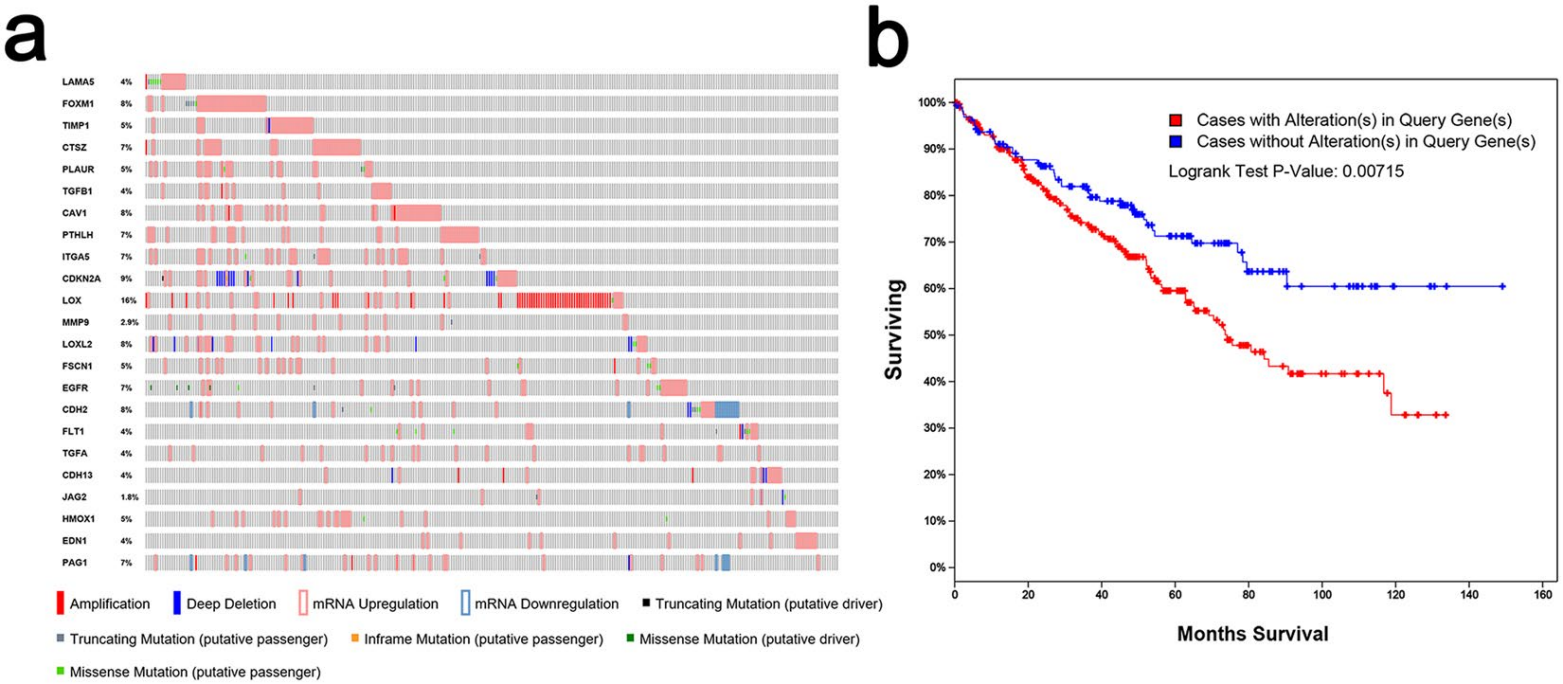
Overview of the main EMT signatures. (**a**) Compact visualization of 23 genomic alterations. The gene set was altered in 285 (63.9%) of 446 complete samples. Genetic alteration included amplification, deep deletion, mRNA upregulation, mRNA downregulation, truncating mutation, and missense mutation. (**b**) Overall survival Kaplan-Meier estimate: Cases with alterations (red): total cases: n = 285, cases deceased: n = 107, median survival in months, 74.11; cases without alterations (blue): total cases: n = 161, cases deceased: n = 43, median survival in months: NA (not available).

Next, CEGs were validated as altered in 285 (64%) of 446 patients in cBioPortal (Fig. 1a). In addition, to test whether the genetic alteration, including amplification, deep deletion, mRNA upregulation and truncating mutation (putative driver), could serve as outcome predictors, the altered signature and clinical data were analysed using the overall survival Kaplan-Meier estimate. As shown in Fig. 1b, cases with alterations in CEGs were correlated with decreased survival (median month survival: 74.11 vs. NA; P = 0.00715). When comparing the separated 14-gene signature and 9-gene signature, only the former showed a significant correlation with prognosis (Supplementary Fig. S1b and S1d). Taken together, these results suggested that the alteration of the 14-gene signature significantly affected the outcome of patients, while changes in the 9-gene signature did not influence the prognosis.

We further explored whether the dysregulation of the 14-gene signature was involved in the development and progression of renal clear cell carcinoma. The clinical data were divided into several subgroups according to the tumour grade, pathologic stage and metastasis. Fourteen mRNAs were demonstrated to be differentially expressed in the histopathologic grade, tumour stage and metastasis subgroups (Supplementary Table S4). Additionally, the distribution of three representative genes, PLAUR, PTHLH and FOXM1, is shown in Supplemental Figs S2 and S3, and their expression was upregulated in the higher grade (P < 0.01), higher clinical stage (P < 0.01), and higher metastasis stage (P < 0.01) merged subgroups, respectively.

### Pathway enrichment analyses in differently expressed genes

**Figure 2:**
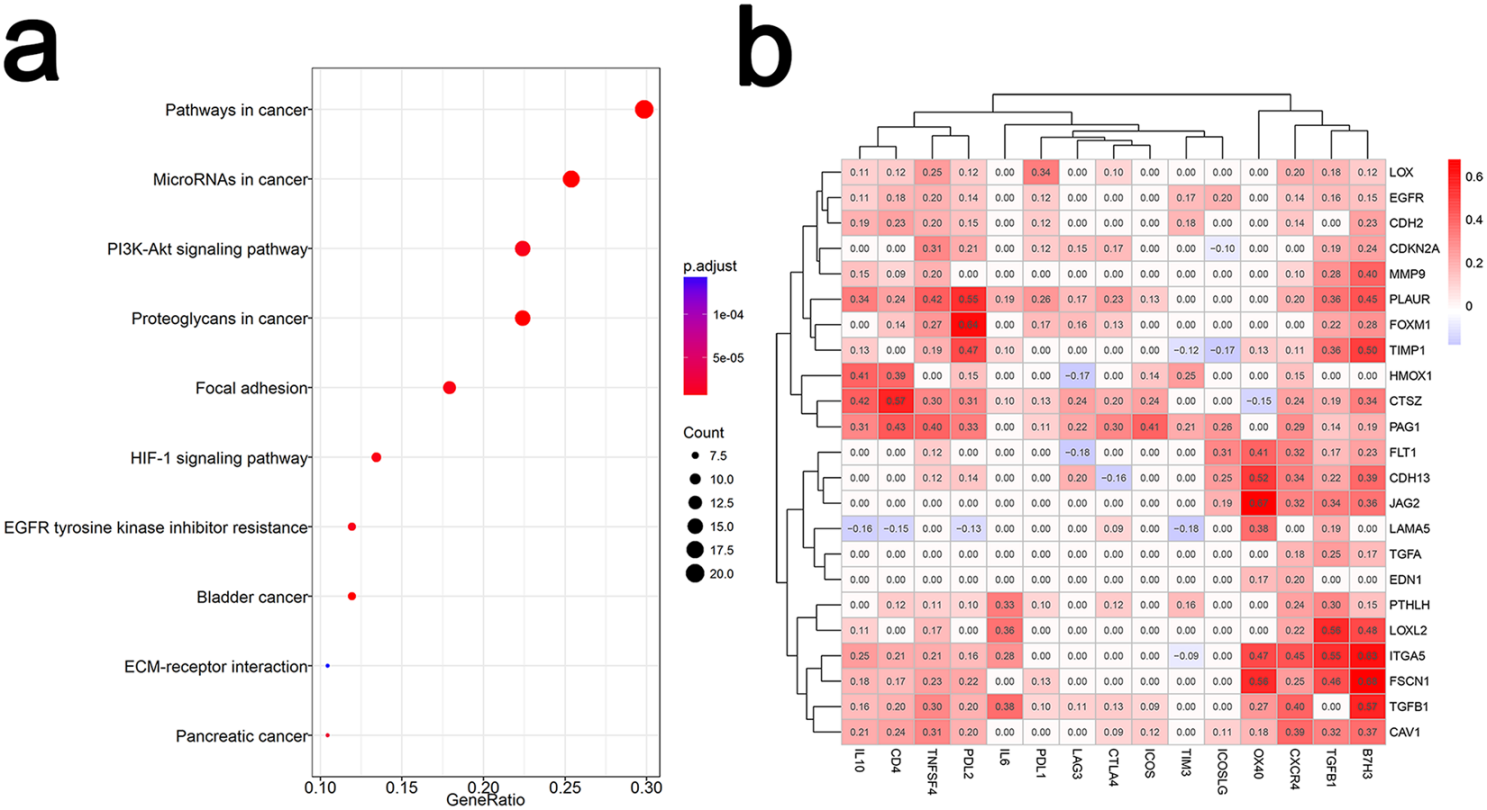
EMT signature-correlated pathways and immune checkpoint molecules. (**a**) Top 10 pathways enriched in the differentially expressed EMT genes. The adjusted P value is shown as dots with different colours. The enrichment count is shown as dots with different sizes. The number of candidate genes in each pathway was calculated as the GeneRatio. (**b**) Correlation of the expression between EMT genes and immune checkpoint genes, based on Pearson correlation coefficients.

To identify the biological functions of the EMT signature in ccRCC, we performed KEGG analysis to identify the enriched pathways. Given that previous research had reported 62 pathways and 28 diseases associated with the entire gene list in dbEMT, our goal was to better understand the extra roles of enriched pathways in 100 dysregulated genes in ccRCC. Interestingly, in addition to cancer-related pathways (e.g., Pathways in cancer, Bladder cancer, Pancreatic cancer) and EMT regulation (e.g., Proteoglycans in cancer, Focal adhesion, ECM-receptor interaction), top several pathways enriched may also be related to immune escape and immune response regulation. Among the top 10 enriched pathways, Proteoglycans in cancer, HIF-1 signalling pathway, and PI3K-Akt signalling pathway were all reported to participate in immune cell signalling in recently reports^13–15^, suggesting a potential relationship between EMT and immunity (Fig. 2a).

### Correlation analysis of selected immune therapeutic targets and CEG

To identify the potential therapeutic targets of EMT and immune escape, 15 representative emerging immune checkpoint genes were selected according to a previous report^11^ and the correlation coefficient in this ccRCC model: IL10, CD4, TNFSF4, PDL2, IL6, PDL1, LAG3, CTLA4, ICOS, TIM3, ICOSLG, OX40, CXCR4, TGFB1, and B7H3 (CD276). The correlation of the expression level between CEG and these genes was analysed, and most of the genes from the two groups were observed to be positively co-expressed. Interestingly, the remarkably correlated immune targets were found to be CD276, OX40, and TGFB1 (median r = 0.35, 0.33, 0.29, median P < 0.001) instead of the well-known PDL1, CTLA4 and TIM3, which had little relationship with the EMT signatures in our model (Fig. 2b, Supplementary Fig. S4a). Additionally, it should be noted that the biological correlation could be direct or indirect. An example of an indirect correlation is that CD276 was reported to promote tumour cell migration and invasion through the Jak2/Stat3/MMP9 signalling pathway^16^, while the r value between CD276 and MMP9 was 0.4 in this analysis.

### Construction and functional analysis of a CEG-immune checkpoint integrated network

**Figure 3:**
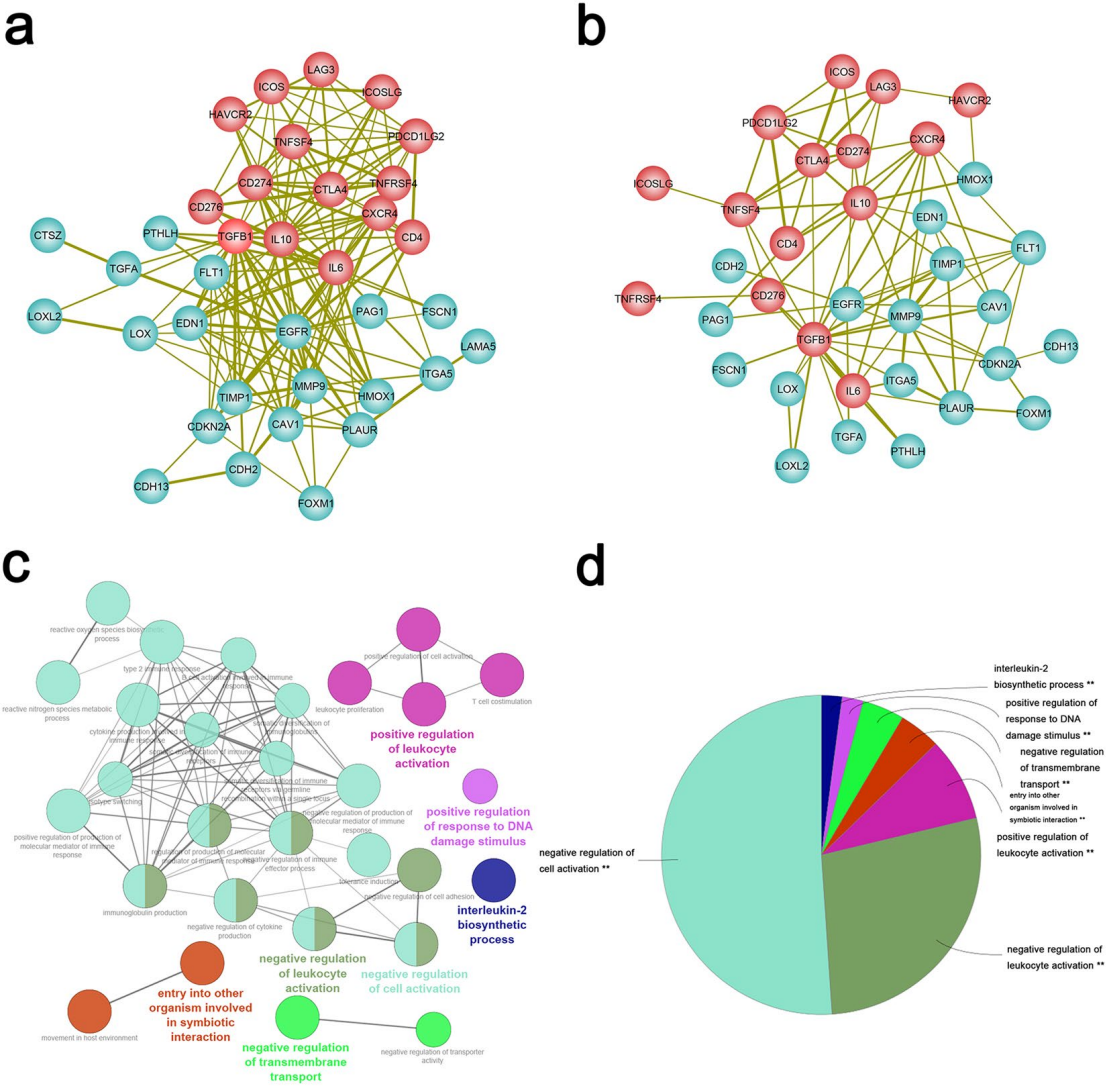
Integrated network and functional annotation. (**a**) EMT-related genes are indicated as cyan circles, immune checkpoint targets are indicated as red circles. The thickness of the lines depends on the combined score in the PPI network. (**b**) A combined network contains both a protein-protein interaction network (PPI) and gene co-expression information. The thickness of the lines depends on the combination coefficient (|co-expression correlation coefficients of mRNAs* combined score in the PPI network|). (**c**) Functional annotation of two correlated signatures. (**d**) Proportion of different functional groups.

To better understand the functional relationships between EMT and immune checkpoints, we integrated data from two different platforms (protein and mRNA). By combining the PPI network and gene co-expression data using a subnetwork extraction algorithm, a common biological network containing both protein and mRNA information was generated. The number of nodes and edges in the PPI network, co-expression network and their common network are shown in Supplementary Table S5 and Fig. 3a, respectively. The network illustrated a significant interaction between two sets of signatures. Among them, TGFB1, CXCR4, IL10, and IL6 represented the most important immune checkpoint molecules interacting with CEG in the integrated network (Fig. 3b).

Finally, we performed gene ontology analysis to determine the functions of the constructed signature. Not surprisingly, the main functions were found to be related to the tumour microenvironment and tumour immune status, such as the negative regulation of cell activation, negative regulation of leukocyte activation, positive regulation of leukocyte activation, entry into other organisms involved in symbiotic interaction, negative regulation of transmembrane transport, positive regulation of response to DNA damage stimulus, and the interleukin-2 biosynthetic process (Fig. 3c,d).

### Evaluation of the clinical significance of CEGs and immune therapeutic target-based approach in patient risk assessment

**Figure 4:**
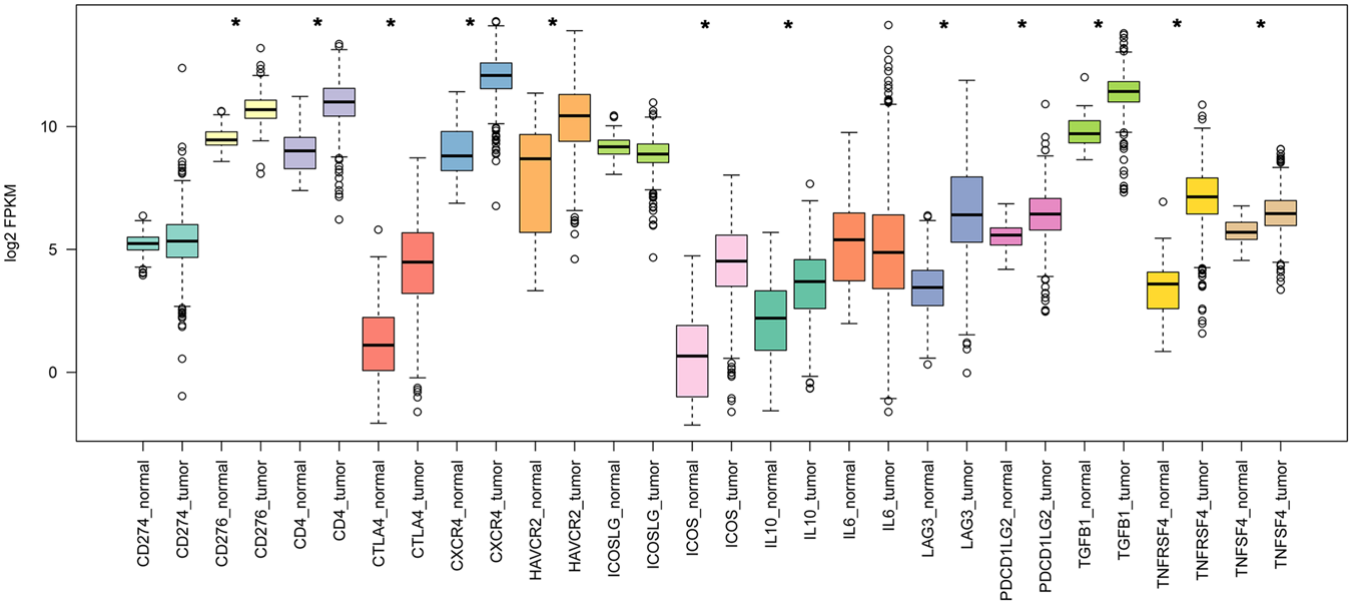
Expression of 15 immune checkpoint genes in 534 tumour samples and 72 adjacent normal samples. “*” indicates a log2FC value >1 and P value < 0.01.

The expression of 15 immune checkpoint genes was then evaluated in the ccRCC data set using a log2KFPM value. Notably, the majority of the targets were expressed more highly in tumours than in normal tissue (Fig. 4). Additionally, the altered immune checkpoint signature was correlated with a poor outcome (P = 0.00536, Supplementary Fig. S4b).

We then established a scoring system to validate the risk assessment abilities of the two associated signatures. First, univariate Cox proportional hazards regression was used, and 15 of 37 genes (including CEG and immune checkpoint genes) were identified to significantly influence the prognosis. Second, the 15 genes were included in a multivariate Cox proportional hazards regression analysis (Supplementary Table S6). Next, three independent risk factors were extracted and exhibited a prognostic signature for ccRCC—FOXM1, TIMP1 and IL6 (Supplementary Table S7).

**Figure 5:**
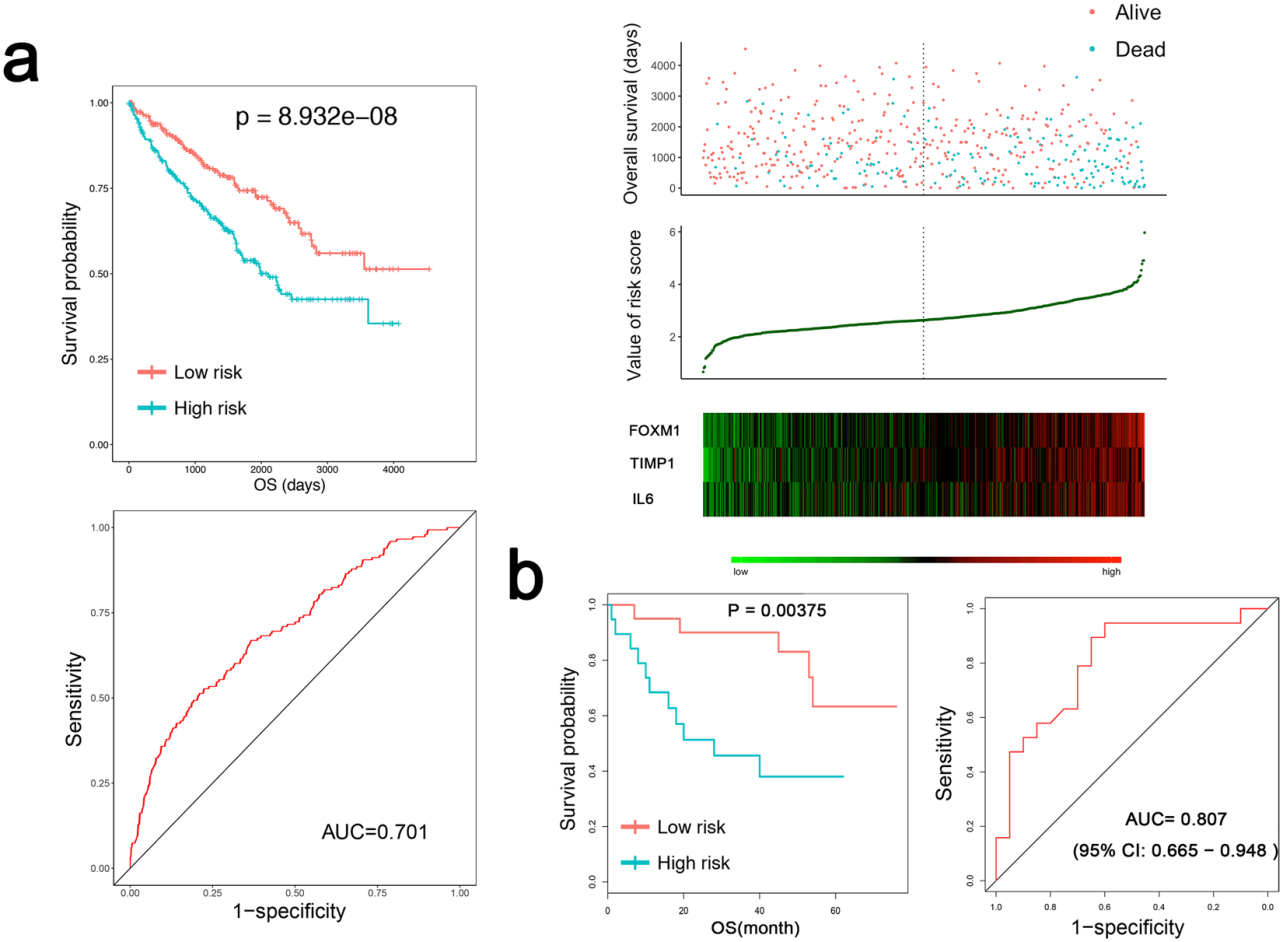
Distinguishing ability of the risk score for the clinical status. (**a**) The Kaplan-Meier test of the risk score for overall survival. P = 8.932e-08. Prognostic performance of the risk score as shown by the time-dependent receiver operating characteristic (ROC) curve for predicting the 5-year survival. The area under the curve (AUC) = 0.701. Patients were divided into high- and low-risk groups using the risk score. The relationship between survival and the risk score is shown at the top; the risk score curve is presented in the middle; heatmap of patients with 3 signatures is on the bottom. (**b**) Validation of the risk score in the GEO dataset (GSE29609). The Kaplan-Meier test of the risk score for overall survival (n = 39, p value = 0.00375). Prognostic performance of the risk score as shown by the time-dependent receiver operating characteristic (ROC) curve for predicting the 5-year survival. Area under the curve (AUC) = 0.807.

The risk score was validated to successfully predict the 5-year survival of ccRCC patients, and the survival time of patients in the high-risk group was predominantly shorter than that in the low-risk group (1252.6 ± 939.6 days vs. 1435.9 ± 1017.3 days, p = 1.4403e-06, Fig. 5a). We further compared the scoring model with other clinical parameters. Age, grade, laterality, pathologic stage, T stage, M stage, N stage and risk score were found to be associated with OS in univariate Cox regression (P < 0.05). Age (>60 years), grade (G3 + G4), M stage (M1) and high-risk score were negatively correlated with OS (P < 0.05) in multivariate Cox regression. The results indicated that a high-risk score was an independent risk factor in ccRCC patients (Table 1).

**Table 1:** Univariate analysis and Multivariate analysis of the risk score.

Variables, n (%)	patient (n = 537)	Univariate analysis HR (95%CI)	*P* ^a^	Multivariate analysis HR (95%CI)	*P* ^b^
**Gender**					
Female	191(35.6)	ref		ref	
Male	346(64.4)	0.949(0.692, 1.302)	0.746	0.893(0.638, 1.251)	0.512
**Age**					
≤60	266(49.5)	ref		ref	
>60	271(50.5)	1.812(1.322, 2.483)	**<0**.**001**	1.603(1.157, 2.22)	**0**.**005**
**Grade**					
G1 + G2	244(45.4)	ref		ref	
G3 + G4	285(53.1)	2.824(1.977, 4.033)	**<0**.**001**	1.618(1.092, 2.395)	**0**.**016**
**Laterality**					
left	253(47.1)	ref		ref	
right	283(52.7)	0.682(0.501, 0.927)	0.015	0.843(0.607, 1.17)	0.307
**Pathologic stage**					
I + II	326(60.7)	ref		ref	
III + IV	208(38.7)	4.196(3.014, 5.843)	**<0**.**001**	1.678(0.786, 3.579)	0.181
**T stage**					
T1 + T2	344(64.1)	ref		ref	
T3 + T4	193(35.9)	3.533(2.577, 4.843)	**<0**.**001**	1.166(0.605, 2.248)	0.647
**M stage**					
M0	426(79.3)	ref		ref	
M1	79(14.7)	4.482(3.265, 6.153)	**<0**.**001**	2.395(1.599, 3.587)	**<0**.**001**
MX	30(5.6)	0.352(0.049, 2.526)	0.299	0.415(0.058, 2.988)	0.382
**N stage**					
N0	240(44.7)	ref		ref	
N1	17(3.2)	3.286(1.747, 6.182)	**<0**.**001**	1.239(0.604, 2.542)	0.559
NX	280(52.1)	0.746(0.543, 1.024)	0.070	0.746(0.532, 1.044)	0.088
**Race**					
white	466(86.8)	ref		—	—
others	64(11.9)	0.675(0.344, 1.325)	0.253	—	—
**Risk score**					
Low risk	370(68.9)	ref		ref	
High risk	156(29.1)	2.711(1.987, 3.697)	**<0**.**001**	1.606(1.139, 2.265)	**0**.**007**

^a^Univariate Cox regression; ^b^Multivariate Cox regression.

**Figure 6:**
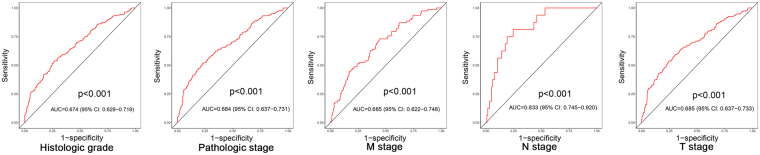
Characteristic (ROC) curve distinguishing the different clinical parameters: grade, clinical stage, clinical M stage, clinical N stage, clinical T stage (P < 0.01).

Furthermore, the prognostic assessment ability of the risk score was further validated using data from the GEO dataset (GSE29609, P = 0.00375) (see Fig. 5b). We also assessed the correlation between the risk score and various clinical parameters, and the risk score showed significant ability to distinguish between the different statuses of the pathological stage (stages I + II vs. Stages III + IV), T stage (T1 + T2 vs. T3 + T4), M stage (M0 vs. M1), N stage (N0 vs. N1) and neoplasm histologic grade (G1 + G2 vs. G3 + G4) in ccRCC (Fig. 6).

## Discussion

Increasing evidence suggests that both the EMT signature and immune checkpoint signature could contribute to tumour progression and metastasis potential. Some EMT-related mRNAs/miRNAs have demonstrated the strength to predict prognosis and other clinical features^9^. However, the complicated functions of the EMT signalling network and its correlation with immune checkpoints in ccRCC remain unclear. Here, we chose a summarized cohort of experimental validated EMT genes to explore the two-signature relationship in ccRCC. We found that in ccRCC patients, 28.01% of EMT genes were dysregulated and 47.34% of EMT genes were significantly correlated with prognosis. In addition, the alteration of CEG was validated to be associated with worse outcomes, and the expression of 14 genes was verified to be positively associated with high histopathologic grade, high tumour stage and metastasis. Notably, the main interactive molecules were discussed in this relationship. Finally, we successfully integrated a risk assessment model based on the EMT signature and immune checkpoint targets.

A major finding in this research is that several targets, such as CD276 and OX40, were shown as key genes co-expressed with the EMT signature. Among these, CD276 is one of the representative novel targets in tumour immunotherapy. In tumour cells, CD276 could reprogram glucose metabolism to benefit growth by ROS-mediated stabilization of HIF-1α^17^. Importantly, Seaman S *et al*. reported that a CD276-targeting ADC could simultaneously target both cancer cells and the tumour vasculature, and the m276-PBD has shown broad tumouricidal and anti-metastatic activity *in vivo*^18^. The underlying strategies include CD276 with blocking monoclonal antibodies (mAbs), CD276-specific antibody-dependent cell-mediated cytotoxicity (ADCC), antibody drug conjugates (ADCs), CD276/CD3 bispecific antibody, CD276-specific small-molecule inhibitor, and CAR T-cell therapy^19^. Furthermore, the latest encouraging results for anti-OX40 therapies in multiple cancer studies indicate the potential great importance of these key genes in kidney cancer^20,21^.

Moreover, HIF-1α is now recognized as a key intermediate between EMT and immune evasion in tumour cells, as summarized in a report from Qiu GZ, *et al*.^22^. HIF-1α-dependent immune evasion through the PI3K/AKT pathway is also important to tumour cells^15^. However, the entire mechanism of tumour immune evasion and immune suppression is far more complicated than we know. The present results indicate the possibility that the EMT signature could participate in this process and be correlated with the tumour immune status in the kidney cancer microenvironment.

The risk scoring model consists of several core genes in cancer-related pathways, EMT-related pathways and immune-related pathways and is verified to be an independent risk factor similar to age, tumour pathological grade and metastasis stage. Based on the current recognition of its complicated biological background, the integrated score provides extra evidence that the two correlated signatures may participate in more malignant biological behaviours and would not be limited to the progression of tumour proliferation and metastasis. These results confirmed that the identification of core molecules in the EMT-immunophenotyping correlation not only provided potential therapeutic targets but also contributed to the prediction of prognosis in ccRCC. At the same time, to further evaluate their efficacy in predicting prognosis and response to immune checkpoint inhibitors, additional prospective clinical follow-up studies would be needed in the future.

In summary, by deeply exploring the correlation between EMT and the immune signature, several emerging targets were found to link or interact with core EMT molecules, and the risk assessment model was verified to successfully predict survival, degree of malignancy and metastasis tendency in ccRCC. The correlated EMT signature provides additional evidence for an optimistic outlook of emerging immune checkpoint blockades and builds confidence for related translational research.

## Methods

### Gene datasets and patient information

We used renal clear cell carcinoma (KIRC) data from the TCGA Data Portal (https://gdc-portal.nci.nih.gov/) and UCSC Xena project (http://xena.ucsc.edu), including RNAseqV2 and clinical data (n = 537). This study meets the publication guidelines provided by TCGA (http://cancergenome.nih.gov/publications/publicationguidelines). The level 3 RNAseq data were derived from 534 ccRCC samples and 72 normal samples. The EMT-associated protein-coding gene list was download from a literature-based resource, dbEMT^12^.

### Public analysis tools

The EMT-related seed genes were analysed by GEPIA, a web server for cancer and normal gene expression profiling and interactive analyses^23^. For the differential expression analysis, one-way ANOVA was used. The genes were first log2(TPM + 1) scaled, and the log2FC was defined as median(Tumour) - median(Normal). The Benjamini and Hochberg false discovery rate (FDR) method was then used to adjust the p-value in each factor to obtain the multiple testing adjusted q-value. Those with |log2FC| > 1 and q value < 0.01 were then considered differentially expressed genes. For the survival analysis, the group cut-off value was the quartile and a logrank P value < 0.01. Furthermore, the candidate genes were verified using cBioPortal for Cancer Genomics^24,25^.

### Functional and signalling pathway analyses

The functional annotation and pathway enrichment analyses for differentially expressed EMT genes and interactive immune checkpoint targets were executed by online analysis tools and ClueGO in cytoscape (version 3.5.1). The KEGG pathway with a p value < 0.05 and a fold enrichment >2.0, and GO terms with a p value < 0.05 and an enrichment score >1.0 were considered significant^26–30^.

### Association between the EMT signature and other covariates

We first applied chi-squared test to assess the association between the candidate EMT signatures and tumour grade, pathologic stage, and metastasis. Next, we used the Wilcoxon test for two groups and the Kolmogorov-Smirnov test for more than two groups to verify the association. In addition, the co-expression relationships between the selected EMT-related genes and immune checkpoints were computed using Pearson’s correlation. The targets without a clear relationship were excluded. All data in tests with P < 0.01 were considered statistically significant.

### Integrated functional network construction

To identify the interaction among the 37 selected genes, we used gene co-expression profiles and the protein-protein interaction network with subnetwork extraction algorithms. The method was reported by Yue *et al*.^31^ and included three steps: (1) First, we built the protein-to-protein network using STRING analysis, and all the interactions with a combined score >0.4 were included^32^; (2) the Pearson co-expression coefficient of 37 genes was computed using R software, and a P value < 0.01 was considered significant. (3) Using the subnetwork extraction algorithm ($${\rm{absolute\; combined\; value}}$$$$=\,{\rm{combined\; score}}\,\times \,{\rm{coexpression\; coefficient}}$$), an integrated network containing both protein and mRNA information was generated by Cytoscape.

### Construction of the EMT and immune checkpoint target-based prognostic signature

The methods for the prognostic model construction were based on previous reports^33,34^. Univariate Cox proportional hazards regression of core EMT and immune checkpoint genes was first performed with a significance level set at 0.05. Based on current recognition of the negative effects of EMT and immune checkpoints in the process of anti-cancer, we excluded genes with a hazard ratio <1, and 15 genes that were negatively correlated with OS (hazard ratio >1; P value < 0.05) were included in the multivariate Cox regression to optimize the prognostic model. Next, the risk score was calculated according to the linear combination of the expression level and regression coefficient using the following formula:

$$\text{risk}\,\text{score}\,=\,ex{p}_{GENE1}\times {\beta }_{GENE1}+ex{p}_{GENE2}\times {\beta }_{GENE2}+\,\cdots \,+ex{p}_{GENEn}\times {\beta }_{GENEn}.$$

(exp represents the expression quantity of the gene, and β represents the regression coefficient derived from the multivariate cox regression model).

Kaplan-Meier survival curves and time-dependent receiver operating characteristic (ROC) curves analysed using R package “survivalROC” were constructed. The survival differences were assessed using the two-sided log-rank test. Patients were divided into high- and low-risk score subgroups according to cut-off values from the ROC method. To evaluate the correlation of the risk score and OS, gender, age, risk score, grade, laterality, pathologic stage, T stage, N stage, M stage and race were included in the univariate Cox proportional hazards regression. Next, variables with a P value < 0.1 (to exclude more influencing factors) and gender were included in the multivariate cox regression. ROC curves were drawn to assess the predictive significance of the prognostic model with clinical features, and the correlation was evaluated using Chi-squared test. Statistical significance was defined as a two-sided P-value less than 0.05.

### Validation of the aberrant expression of candidate genes and clinical value of the risk score in ccRCC based on GEO datasets

Data from the Gene Expression Omnibus database (GEO, http://www.ncbi.nlm.nih.gov/geo) were analysed. One original study that contained gene expression profiling data was collected (GSE53757). The SAM method was used to evaluate the levels of differentially expressed genes between human ccRCC tissues and normal tissues, with a cut-off fold change >1.5, p-value < 0.05 and false discovery rate q-value < 0.05. The Kaplan-Meier survival curves and ROC curve analysis were used to validate the predictive value of the risk score for ccRCC patients based on the GEO dataset (GSE29609).

### Data Availability

The datasets analysed during the current study are available in the TCGA Data Portal (https://gdc-portal.nci.nih.gov/), GEO datasets (https://www.ncbi.nlm.nih.gov/gds/) and dbEMT repository (http://dbemt.bioinfo-minzhao.org/).

## Electronic supplementary material

Supplementary Information
