# Supplementary material for: The Correlation Between the Immune and Epithelial-Mesenchymal Transition Signatures Suggests Potential Therapeutic Targets and Prognosis Prediction Approaches in Kidney Cancer
Source: Sci Rep. 2018 Apr 26;8:6570. doi: 10.1038/s41598-018-25002-w (PMC5919934; doi:10.1038/s41598-018-25002-w)
Supplement: Supplementary file 1 — Supplementary Information [file 41598_2018_25002_MOESM1_ESM.doc]

The Correlation Between the Immune and Epithelial-Mesenchymal Transition Signatures Suggests Potential Therapeutic Targets and Prognosis Prediction Approaches in Kidney Cancer

Jiayu Liang^1^, Zhihong Liu^1^, Zijun Zou^1^, Yongquan Tang^1^, Chuan Zhou^1^, Jian Yang^2^,

Xin Wei^1^ and Yiping Lu^1^


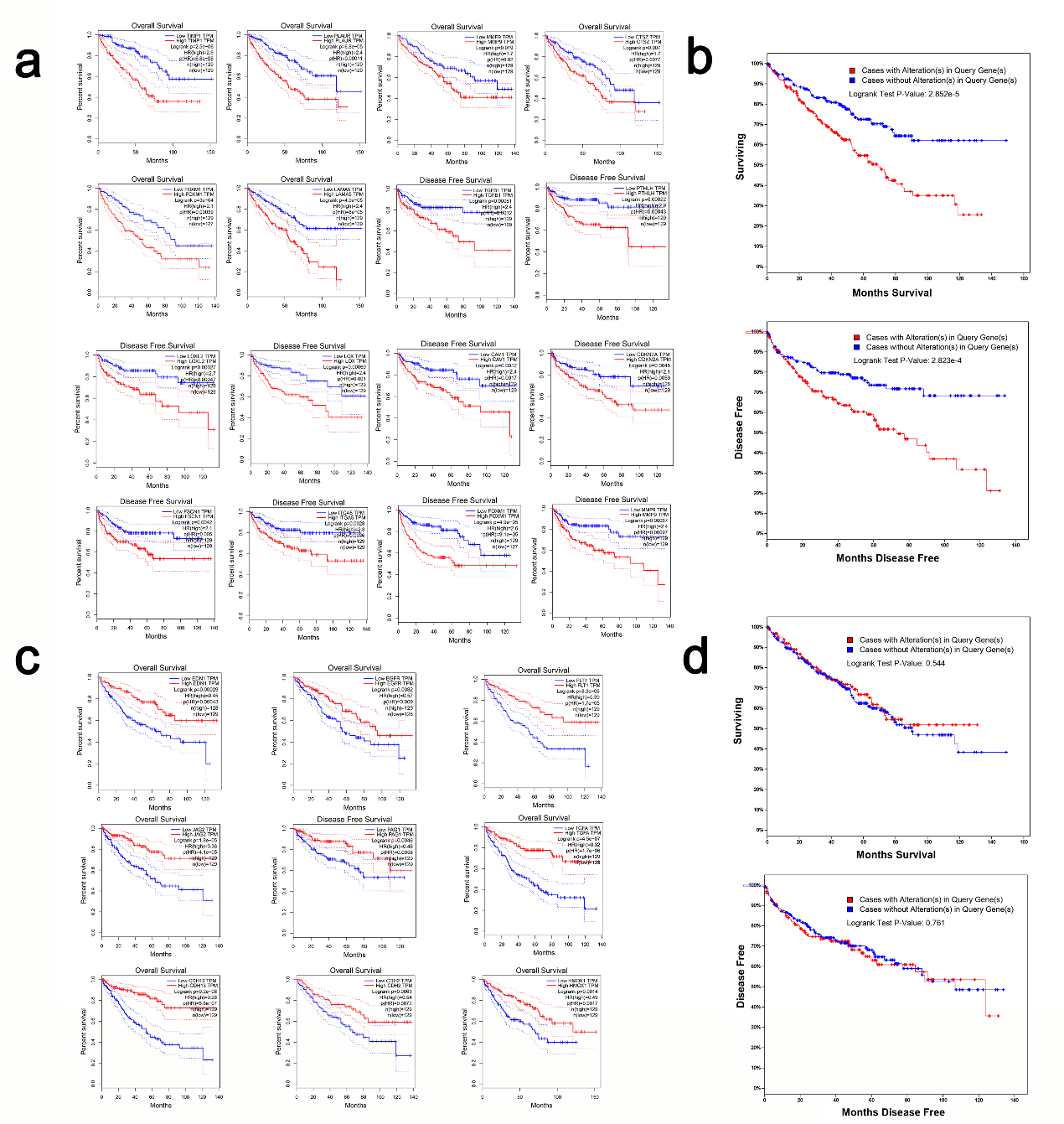
 Supplementary Figure S1. survival plots of candidate genes. **a** Overall survival and disease-free survival plots of 14 genes by GEPIA analyses (Logrank P<0.01). **b** Survival plots of 14-merged gene by cBioPortal analyses (Logrank Test P-value<0.01). **c** Overall survival and disease-free survival plots of 9 genes by GEPIA analyses (Logrank P<0.01). **d** Survival plots of 9-merged signature by cBioPortal analyses (Logrank Test P-value>0.05).


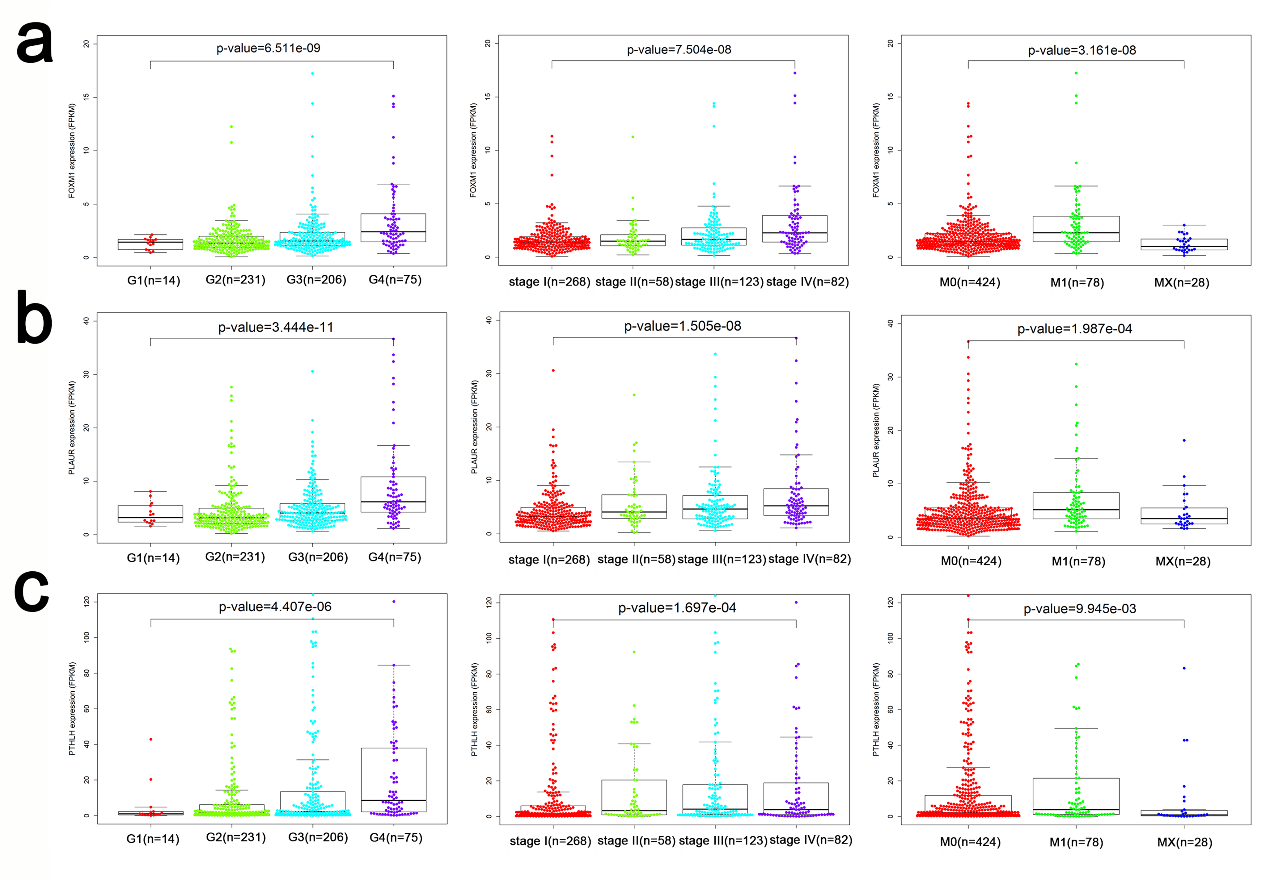


Supplementary Figure S2. Kolmogorov-Smirnov tests of three genes. **a,b,c** The average expression levels of FOXM1, PLAUR and PTHLH in each subgroup of pathological grades, clinical stages and metastasis, respectively (P<0.01).


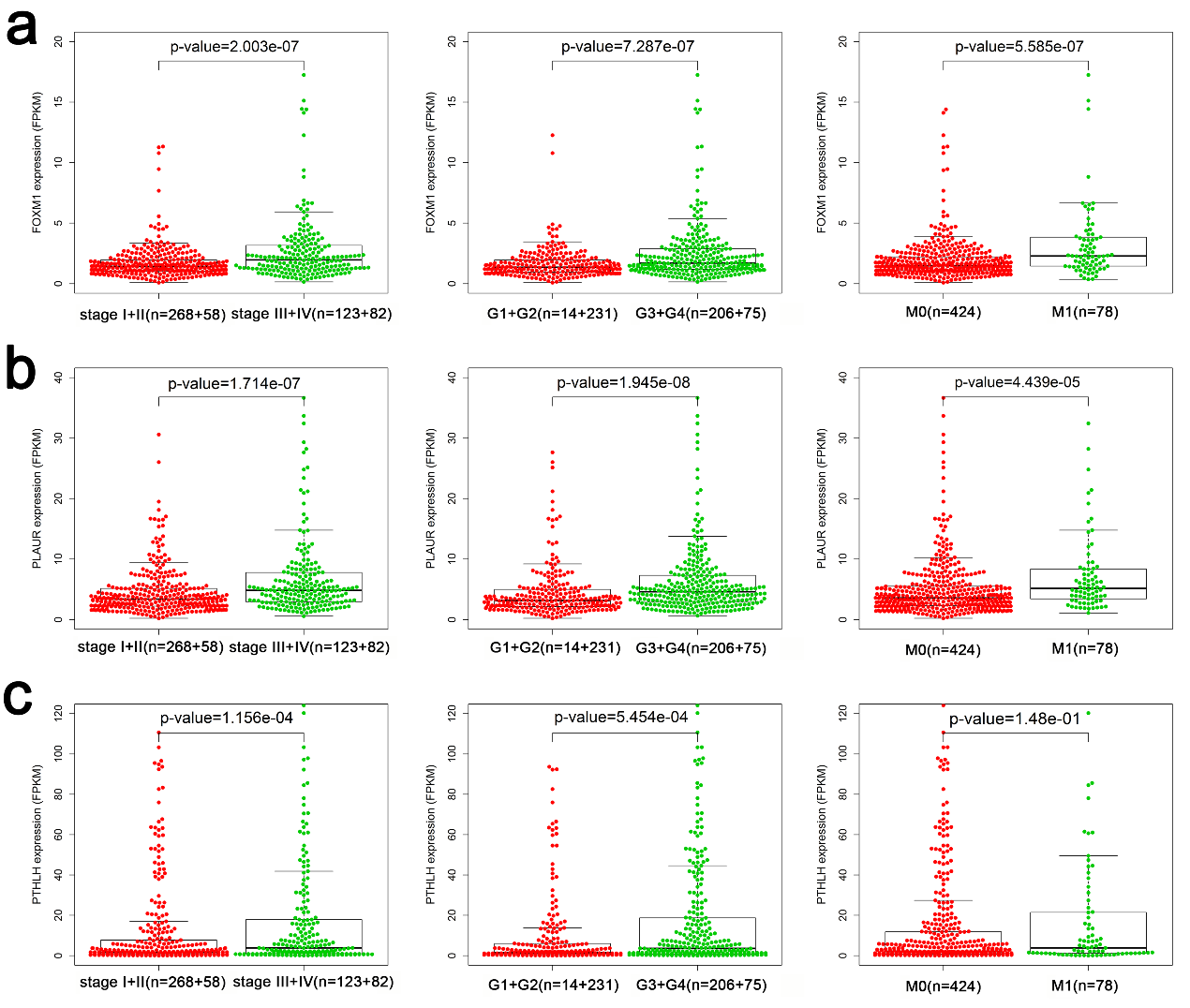


Supplementary Figure S3. Wilcoxon tests of three genes. **a,b,c** The average expression levels of FOXM1, PLAUR and PTHLH in each merged subgroup of pathological grades, clinical stages and metastasis, respectively (P<0.01).


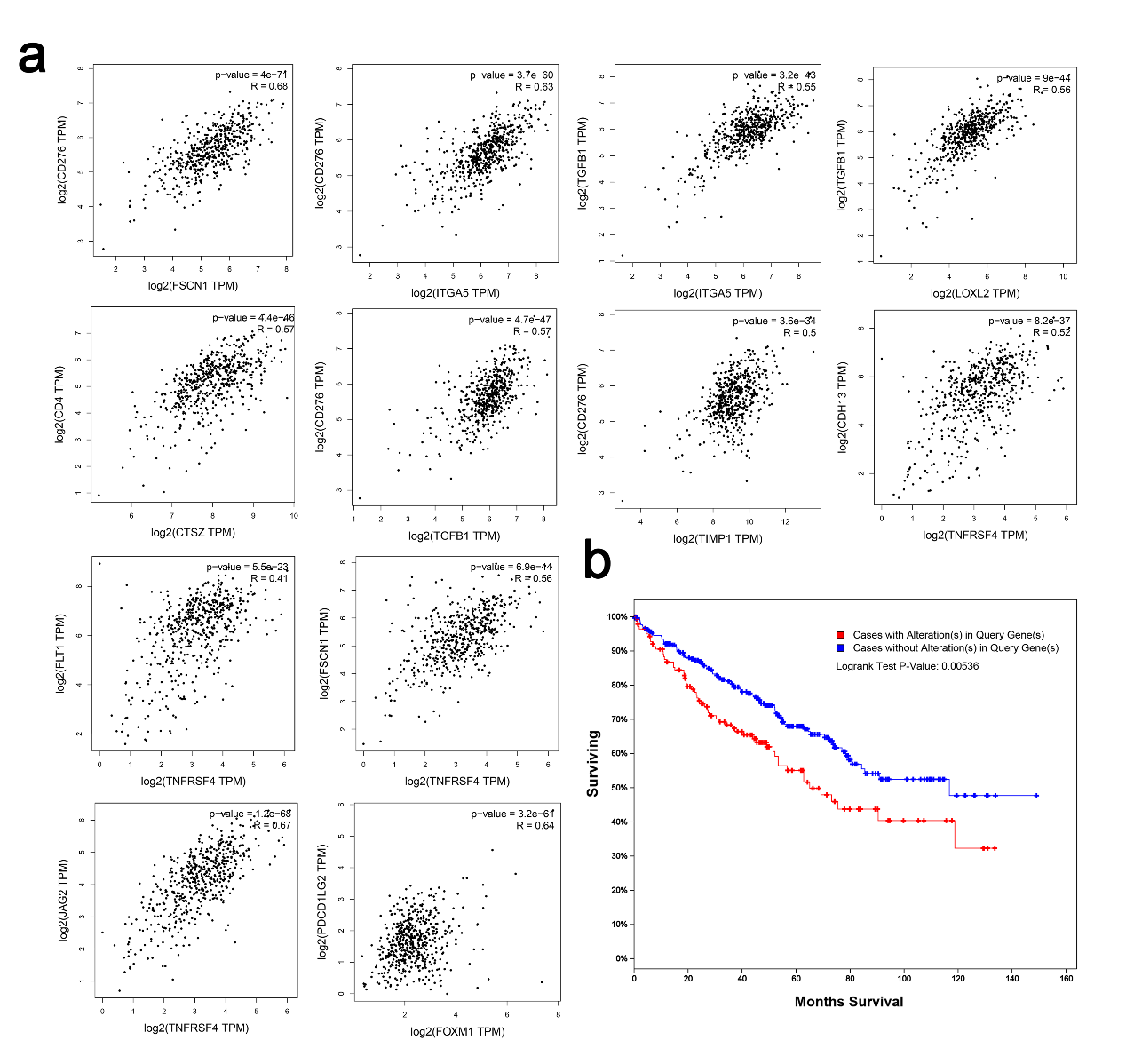


Supplementary Figure S4. Overview of 15 immune checkpoint genes. **a** pair-wise gene expression correlation plots of genes from two groups, testing by Pearson correlation method(P<0.01). **b** Survival plots of 15 immune checkpoint signature by cBioPortal analysis (Logrank Test P-value<0.01)

Supplementary Table S1 Clinical features

| Clinical features | N=537 |  |  |  |
| --- | --- | --- | --- | --- |
| Age  years mean (range) | 61（26-90） |  |  |  |
| Gender(no.)  male, female | 346 | 191 |  |  |
| Pathological T stage  no  T1，T2，T3，T4 | 275 | 69 | 182 | 11 |
| Pathological N stage  no.  N0，N1，Nx | 240 | 17 | 280 |  |
| Pathological M stage no.  M0, M1，Mx | 426 | 79 | 30 |  |
| Pathological grade  no.  G1，G2，G3，G4 | 14 | 230 | 207 | 78 |
| AJCC TNM stage,  no.  I, II, III, IV | 269 | 57 | 125 | 83 |

Supplementary Table S2 Differentially expressed and prognostic EMT-associated genes

| Differentially expressed genes | Up-regulated  (n=46) | **TGFB1, EGFR, LAMA5, CDH2, MYC, FN1, AXL, CAV1, FLT1, CD44, PTHLH, TGM2, RUNX3, VEGFA, ANXA1, FOXM1, ITGA5, VIM, CXCR4, VCAM, CDKN2A, TGFA, TIMP1, MCAM, NDRG1, NRP2, CTSZ, CDH13, IGFBP3, JAG2, MMP14, PAG1, PLXND1, EZH2, LOX, EDNRA, PLAUR, MMP9, POSTN, POU5F1, HMOX1, EDN1, LOXL2, FSCN1, COL8A1, VWCE** |
| --- | --- | --- |
|  | Down-regulated  (n=54) | **CDH1, ERBB2, EGF, ITGB3, TNC, WT1, SIM2, PRKCA, EGR1, KIT, VDR, FGFR2, TBX3, TGFBR3, HBEGF, GATA3, PKP3, TCF21, L1CAM, MUC1, CTGF, WISP3, FBLN5, KRT19, HSP90AA1, PTGS2, MMP7, ST14, ETV4, CLDN4, HPGD, TDGF1, HOXB7, EPB41L5, BMP7, CXCL12, PROM1, FOXC1, FGF1, PRSS8, KL, HOXB9, SDC1, OCLN, VSNL1, TMPRSS4, LCN2, CBR1, KLK6, ELF5, AGER, IDH2, MARVELD3, HS6S72** |
| Prognostic genes(OS) | Positive  (n=119) | **CTNNB1, SMAD3, EGFR, CDH1, ILK, ZEB1, EPAS1, SMAD2, SMAD4, IGF1R, ERBB2, AKT1, CTNND1, NFKB1, CTNNBIP1, CDH2, TCF4, YY1, ACVR1, KLF4, ERBB21P, SP1, TGFB2, KLF6, MAPK1, GSK3B, PIK3CA, KHDRBS1, MAPK14, DAB2, KIT, ITGA6, STAT5B, BMP2, PTK2, TGFBR1, ID2, PPARG, FLT1, YAP1, TGFBR3, NUMB, JAG1, NOTCH1, PRKCE, NF1, CDKN1B, ENG, MAPK3, AR, KRAS, HNF4A, GAB2, PTEN, ROCK1, GSN, MYCN, BMI1, TCF21, NFIC, ROCK2, MSN, BRAF, TGFA, TEAD1, JAK2, LRP6, MAPK8, NDRG1, ZBTB33, HMGB1, RHOA, PTPN14, RNF111, SNW1, BCL2, CDH13, WWTR1, PAK1, RDX, ZNF217, PDGFB, JAG2, DNAJB6, HSP90AA1, ZFYVE9, MKL2, CLDN4, PIN1, LIMA1, HPGD, EPB41L5, PHLDA1, ANPEP, DAPK1, CLU, LATS1, HMOX1, EDN1, PCMT1, IL6R, SEMA4C, MTDH, PRSS8, CMTM8, KL, TXNIP, TM4SF5, MUC4, BCL2L1, GIPC2, LETMD1, OCLN, SDC2, CAMK1D, PDE4A, CD274, GLRX, MARVELD3** |
|  | Negative  (n=27) | **LAMA5, ITGB4, WT1, SIM2, FOXA1, PKP3, FOXM1, MDK, MSX2, HMGA2, TIMP1, CTSZ, VTN, ROR2, FSCN2, KRT19, PLAUR, GREM1, SCRIB, BOP1, DLX4, HOXB9, VSNL1, CXCL5, LCN2, WISP2, AGER** |
| Prognostic genes(RFS) | Positive  (n=59) | **EPAS1, SMAD4, IGF1R, ERBB2, AKT1, CTNND1, NFKB1, KLF4, ERBB2IP, KLF6, MAPK1, GSK3B, ITGA6, STAT5B, PTK2, PPARG,TGFBR3, NUMB, PRKCE, MAPK3, AR, KRAS, GAB2, ESR1, ROCK1, TRPS1, DDX5, BRAF, TEAD1, JAK2, MAPK8, ZBTB33, RHOA, PAX2, RNF111, BCL2, RDX, ZNF217, MAP3K4, PAG1, ZFYVE9, CLDN4, LIMA1, HPGD, EPB41L5, DAPK1, LATS1, PCMT1, PRSS8, KL, TXNIP, MUC4, BCL2L1, GIPC2, OCLN, SDC2, CBR1, CD274, MARVELD3** |
|  | Negative  (n=28) | **TGFB1, WT1, CAV1, PTHLH, FOXA1, PKP3, FOXM1, ITGA5, HSPB1, MSX2, CDKN2A, FIMP1, ROR2, KRT19, SEMA7A, LOX, PLAUR, MMP9, LEFTY1, NCSTN, BOP1, DLX4, LOXL2, FSCN1, IL6R, MAGED1, CXCL5, WISP2** |
| Overlapped genes | Up-regulated/ Negative  (n=14) | **TGFB1, LAMA5, PTHLH, FOXM1, TIMP1, CAV1, CDKN2A, ITGA5, CTSZ, LOX, PLAUR, MMP9, LOXL2, FSCN1** |
|  | Up-regulated/ Positive  (n=9) | **EGFR, CDH2, FLT1, TGFA, CDH13, JAG2, PAG1, HMOX1, EDN1** |

Supplementary Table S3 Validation of expression of 23 candidate genes in ccRCC based on GEO dataset GSE53757

|  | N  (tumor) | Mean  (tumor) | N  (normal) | Mean  (normal) | d score | Fold Change | p-value | q-value |
| --- | --- | --- | --- | --- | --- | --- | --- | --- |
| LAMA5 | 72 | 8.934716 | 72 | 8.259447 | 10.287467 | 1.596894 | <0.05 | <0.05 |
| FOXM1 | 72 | 5.0897 | 72 | 4.127217 | 14.071473 | 1.94866 | <0.05 | <0.05 |
| TIMP1 | 72 | 11.580188 | 72 | 9.481314 | 14.373521 | 4.283749 | <0.05 | <0.05 |
| CTSZ | 72 | 8.335616 | 72 | 7.159212 | 10.49511 | 2.260128 | <0.05 | <0.05 |
| PLAUR | 72 | 7.627144 | 72 | 6.609719 | 8.114564 | 2.024304 | <0.05 | <0.05 |
| TGFB1 | 72 | 8.452355 | 72 | 6.558672 | 15.684093 | 3.715825 | <0.05 | <0.05 |
| CAV1 | 72 | 10.861214 | 72 | 7.682328 | 22.59371 | 9.056077 | <0.05 | <0.05 |
| PTHLH | 72 | 5.493887 | 72 | 2.554696 | 8.936979 | 7.669809 | <0.05 | <0.05 |
| ITGA5 | 72 | 8.260289 | 72 | 6.497141 | 20.448588 | 3.394382 | <0.05 | <0.05 |
| CDKN2A | 72 | 4.563448 | 72 | 3.259129 | 14.835087 | 2.469672 | <0.05 | <0.05 |
| LOX | 72 | 10.445642 | 72 | 6.118423 | 20.924136 | 20.073481 | <0.05 | <0.05 |
| MMP9 | 72 | 6.148324 | 72 | 5.124523 | 7.141604 | 2.033269 | <0.05 | <0.05 |
| LOXL2 | 72 | 8.906143 | 72 | 5.556995 | 23.272048 | 10.190463 | <0.05 | <0.05 |
| FSCN1 | 72 | 6.118581 | 72 | 4.667418 | 15.084577 | 2.734283 | <0.05 | <0.05 |
| EGFR | 72 | 10.559734 | 72 | 8.573673 | 15.2236 | 3.961538 | <0.05 | <0.05 |
| CDH2 | 72 | 9.271808 | 72 | 8.598774 | 6.588898 | 1.594422 | <0.05 | <0.05 |
| FLT1 | 72 | 5.7581 | 72 | 3.843178 | 15.392432 | 3.770935 | <0.05 | <0.05 |
| TGFA | 72 | 9.658915 | 72 | 7.687606 | 22.690552 | 3.921238 | <0.05 | <0.05 |
| CDH13 | 72 | 7.555773 | 72 | 5.772674 | 14.75662 | 3.441646 | <0.05 | <0.05 |
| JAG2 | 72 | 7.52107 | 72 | 6.077488 | 16.383536 | 2.719955 | <0.05 | <0.05 |
| HMOX1 | 72 | 9.826511 | 72 | 8.390599 | 11.764097 | 2.70553 | <0.05 | <0.05 |
| EDN1 | 72 | 5.856501 | 72 | 4.463639 | 10.012565 | 2.625992 | <0.05 | <0.05 |
| PAG1 | 72 | 7.451726 | 72 | 5.160865 | 22.813071 | 4.893478 | <0.05 | <0.05 |

Supplementary Table S4 Clinical covariates correlation of 14 up-regulated and poor survival associated genes

|  | Pathologic  stage | Pathology T  stage | Pathology N  stage | Pathology M  stage | Histopathologic grade |
| --- | --- | --- | --- | --- | --- |
| CTSZ | 8.10E-62 | 3.75E-214 | 1.65E-89 | 1.60E-208 | 5.66E-126 |
| LOX | 9.86E-68 | 5.29E-176 | 9.78E-85 | 5.31E-191 | 3.31E-121 |
| PLAUR | 9.84E-77 | 1.35E-175 | 6.21E-88 | 1.38E-197 | 5.81E-129 |
| CAV1 | 4.09E-97 | 5.08E-188 | 4.15E-93 | 1.94E-200 | 4.06E-117 |
| MMP9 | 2.70E-85 | 1.39E-176 | 1.44E-80 | 9.53E-183 | 1.51E-137 |
| FOXM1 | 2.03E-106 | 8.31E-197 | 6.62E-89 | 8.05E-195 | 4.40E-140 |
| CDKN2A | 2.24E-105 | 8.08E-184 | 3.38E-84 | 4.40E-197 | 1.79E-126 |
| LOXL2 | 5.08E-100 | 2.69E-169 | 4.06E-86 | 4.98E-189 | 2.98E-125 |
| TGFB1 | 3.14E-139 | 4.78E-202 | 2.62E-91 | 2.99E-204 | 6.08E-117 |
| PTHLH | 6.63E-91 | 4.93E-167 | 8.42E-81 | 5.31E-186 | 5.05E-133 |
| LAMA5 | 8.27E-121 | 4.24E-180 | 1.02E-91 | 1.82E-202 | 4.01E-119 |
| TIMP1 | 1.33E-125 | 1.29E-192 | 3.63E-86 | 1.51E-194 | 6.57E-127 |
| ITGA5 | 2.16E-131 | 4.61E-199 | 1.48E-89 | 8.03E-201 | 6.20E-124 |
| FSCN1 | 1.15E-127 | 8.69E-177 | 2.51E-95 | 3.49E-196 | 1.66E-121 |

Supplementary Table S5 PPI network and co-expression pairs

|  | PPI | CO-EXP | COMMON |
| --- | --- | --- | --- |
| Nodes | 36 | 37 | 34 |
| Edges | 155 | 377 | 88 |

Supplementary Table S6 Prognostic value of 15-signature by univariate and multivariate analysis

|  | Univariate analysis | | | Multivariate analysis | | |
| --- | --- | --- | --- | --- | --- | --- |
|  | HR 95% CI | | p-value | HR 95% CI | | p-value |
| FOXM1 | 1.606 | 1.406-1.835 | 2.85E-12 | 1.541 | 1.305-1.820 | 3.35E-07 |
| TIMP1 | 1.548 | 1.3543-1.771 | 1.83E-10 | 1.454 | 1.180-1.792 | 0.000433657 |
| IL6 | 1.447 | 1.271-1.646 | 2.05E-08 | 1.110 | 1.022-1.207 | 0.013240525 |
| CD276 | 1.370 | 1.192-1.575 | 9.43E-06 | 0.665 | 0.410-1.079 | 0.098683426 |
| PLAUR | 1.151 | 1.075-1.233 | 6.00E-05 | 1.155 | 0.918-1.451 | 0.218101531 |
| LOXL2 | 1.190 | 1.081-1.309 | 0.000375171 | 0.878 | 0.712-1.083 | 0.224785897 |
| LAG3 | 1.147 | 1.060-1.242 | 0.000695318 | 0.930 | 0.815-1.061 | 0.280199918 |
| CTLA4 | 1.261 | 1.095-1.451 | 0.001248929 | 1.081 | 0.927-1.259 | 0.314491374 |
| FSCN1 | 1.354 | 1.121-1.636 | 0.001650755 | 1.098 | 0.865-1.393 | 0.444489382 |
| MMP9 | 1.314 | 1.090-1.585 | 0.004243523 | 0.972 | 0.877-1.077 | 0.582097858 |
| TNFSF4 | 1.324 | 1.086-1.615 | 0.005572973 | 1.065 | 0.835-1.359 | 0.609958765 |
| TGFB1 | 1.080 | 1.021-1.141 | 0.006737809 | 0.943 | 0.687-1.295 | 0.717823757 |
| CDKN2A | 1.404 | 1.083-1.820 | 0.010354762 | 1.019 | 0.880-1.180 | 0.803907799 |
| CTSZ | 1.245 | 1.051-1.474 | 0.010992293 | 0.985 | 0.761-1.274 | 0.907645803 |
| PTHLH | 1.155 | 1.011-1.320 | 0.033713397 | 1.001 | 0.934-1.073 | 0.976433185 |

Supplementary Table S7 Multivariate analysis of three independent risk factors

|  | coef | HR. | lower..95 | upper..95 | z | Pr...z.. |
| --- | --- | --- | --- | --- | --- | --- |
| FOXM1 | 0.365627 | 1.441418 | 1.252358 | 1.65902 | 5.096861 | 3.45E-07 |
| TIMP1 | 0.240095 | 1.27137 | 1.09419 | 1.477242 | 3.13549 | 0.001716 |
| IL6 | 0.06986 | 1.072358 | 1.00608 | 1.143001 | 2.146194 | 0.031857 |
